# The rule of brain hematoma pressure gradient and its influence on hypertensive cerebral hemorrhage operation

**DOI:** 10.1038/s41598-021-84108-w

**Published:** 2021-02-25

**Authors:** Guoqing Sun, Tingkai Fu, Zhaoyan Liu, Yuhai Zhang, Xiangtao Chen, Shigang Jin, Feng Chi

**Affiliations:** Department of Neurosurgery, Rizhao People’s Hospital Affiliated With Jining Medical University, Rizhao, 276826 Shandong Province People’s Republic of China

**Keywords:** Stroke, Neurodegeneration

## Abstract

To comparatively study the size of and variation in the ‘brain-haematoma’ pressure gradient for different surgical methods for hypertensive intracerebral haemorrhage (HICH) and analyse the gradient’s influence on surgical procedures and effects of the haemorrhage. Seventy-two patients with HICH treated from 1/2019 to 12/2019 were randomly divided into two groups, namely, the keyhole endoscopy and large trauma craniotomy groups, according to different operative methods. Intraoperative changes in intracranial pressure (ICP) were monitored to calculate intraoperative alterations in the ‘brain-haematoma’ pressure gradient. Intraoperative characteristics (operative time, bleeding volume, volume of blood transfusion, and haematoma clearance rate) and postoperative characteristics (oedema, postoperative activities of daily living (ADL) scores, mortality rate and rebleeding rate) were compared between the two groups. In the keyhole endoscopy group, ICP decreased slowly; the ‘brain-haematoma’ pressure gradient was large, averaging 251.1 ± 20.6 mmH_2_O, and slowly decreased. The mean operative time was 83.6 ± 4.3 min, the mean bleeding volume was 181.2 ± 13.6 ml, no blood transfusions were given, the average postoperative haematoma clearance rate was 95.6%, the rate of severe oedema was 10.9%, and the average postoperative ADL score was 85.2%. In the large trauma craniotomy group, ICP rapidly decreased after craniotomy. When the haematoma was removed, the ‘brain-haematoma’ pressure gradient was small, averaging 132.3 ± 10.5 mmH2O, and slowly decreased. The mean operative time was 232 ± 26.1 min, the mean bleeding volume was 412.6 ± 35.2 ml, the average volume of blood transfusion was 281.3 ± 13.6 ml, and the average postoperative haematoma clearance rate was 82.3%; moreover, the rate of severe oedema was 72.1%, and the average postoperative ADL score was 39.0%. These differences were statistically significant (*P* < 0.05). Neither the death rate (*P* > 0.05, 2.7% VS 2.8%) nor rebleeding rate (*P* > 0.05, 2.7% VS 2.8%) showed any obvious changes. The magnitude and variation in the ‘brain-haematoma’ pressure gradient for different surgical methods significantly influence surgical procedures and effects of HICH. During keyhole endoscopy surgery, this gradient was relatively large and slowly decreased; the haematoma was therefore easier to remove. Advantages of this approach include a high haematoma clearance rate, decreased bleeding volume, decreased operative time, reduced trauma, decreased postoperative brain oedema and improved postoperative recovery of neurological function.

*Chinese Clinical Trial Register*: ChiCTR1900020655 registration in 12/01/02,019 registration in 28/02/02,020 Number: NCOMMS-20–08,091.

## Introduction

Hypertensive intracerebral haemorrhage (HICH) is a common neurosurgical condition with high morbidity^1^ that accounts for 70% of stroke patients^2^ and is associated with high mortality and a high disability rate^3^; in particular, 61–88% of patients become severely disabled^4,5^. The ‘brain-haematoma’ pressure gradient plays an important role in the process of cerebral haemorrhage and in surgery. Variations in this gradient differ under three distinct conditions: post-cerebral haemorrhage, keyhole endoscopy and craniotomy. Changes in the “brain-haematoma” pressure gradient determine movement direction for the brain and haematoma, and differences in these changes in various operations produce diverse effects on surgical procedures and postoperative results. We studied 72 patients with intracerebral haemorrhage who were treated from 1/2019–12/2019. In addition, for summary and analysis, we compared changes in the ‘brain-haematoma’ pressure gradient and its influence on surgery and postoperative effects for HICH patients treated with two different types of operations.

## Materials and methods

### Ethics

This study was approved by the Medical Ethical Committee of Rizhao People’s Hospital Affiliated with Jining Medical University. This research was performed in accordance with relevant guidelines and regulations, and informed consent was obtained from all participants or their legal guardians.

### Clinical materials

The patients in the two groups were diagnosed with HICH from 1/2019 to 12/2019 and had a haematoma that was locatable by imaging with a volume greater than 30 ml, all haematoma located above the tentorium, and they all had a history of hypertension. Patients with cerebral haemorrhage caused by intracranial aneurysm, intracranial arteriovenous malformation, tumours, haemorrhage after cerebral infarction, long-term use of anticoagulants, and other disorders were excluded, as were cerebral hernia patients and patients with severe systemic diseases or dysfunction of other important organs, such as the heart, lung, liver and kidney. According to operative method, the patients were randomly divided into two groups: the keyhole endoscopy group and the large trauma craniotomy group by random scale. Both groups of patients were treated within 24 h after paroxysm. There were 36 patients in the keyhole endoscopy group, including 19 males and 17 females, with an average age of 48.2 ± 6.1 years (range, 31 to 72 years). The preoperative haematoma volume was 46.2 ± 5 ml, and the Glasgow Coma Scale (GCS) score was 7.7 ± 2.1. Seventeen, 13 and 6 patients had haemorrhage in the basal ganglia, capsula externa, and brain lobe, respectively. There were 36 patients in the large trauma craniotomy group, including 21 males and 15 females, aged 47.9 ± 6.5 years (range, 36 to 67 years). The preoperative haematoma volume was 47.8 ± 5.6 ml, and the GCS score was 7.6 ± 3.1. There were 19, 8 and 9 cases of haemorrhage in the basal ganglia, capsula externa, and brain lobe, respectively. The general preoperative conditions of the two groups of patients were compared, and there was no significant difference between the groups (*P* > 0.05), meaning that they were comparable.

### Calculation of the ‘brain-haematoma’ pressure gradient

Both groups of patients received intracranial pressure (ICP) monitoring before undergoing craniotomy. After general anaesthesia, all patients underwent puncture of the anterior corner of the lateral ventricle and implanted intracranial pressure sensor (ventricular catheter type, model 826653, American Johnson & Johnson Company, CODMAN). The site was the frontal puncture point (2.5 cm before the coronal suture and 2.5 cm beside the midline). The skull was drilled, the dura was cut, the brain was punctured into the lateral ventricle, and the sensor was implanted at a depth of 6.0–7.0 cm. Next, the needle core was removed, the cerebrospinal fluid overflow was visualized, an ICP was placed to monitor the lateral ventricle, and the probe lead of the ICP monitor was extended through the subcutaneous tunnel. During the operation, the probe was continuously fixed in the ventricle. After the intracranial pressure monitor was zeroed during the operation, the intracranial pressure was continuously monitored throughout the operation. Postoperative ICP monitoring was continued, and cerebrospinal fluid drainage was performed for 3–10 days, with an average monitoring of 7.2 days.

The measured ICP was the brain tissue pressure. Data on the craniotomy were recorded, as were intraoperative and immediately postoperative data. For the calculation of the haematoma pressure, the haematoma was located in the brain tissue before and after the craniotomy, such that the pressure in the haematoma cavity and the brain tissue pressure were in a dynamic equilibrium, meaning the pressure values were equal. At this time, the ‘brain-haematoma’ pressure gradient = ICP–ICP = 0 mmH_2_O. When the haematoma was removed, the haematoma cavity became connected to the external environment, the value of the haematoma cavity pressure returned to 0 mmH_2_O, and the ‘brain-haematoma’ pressure gradient = ICP–0 = ICP. Values of ICP and the ‘brain-haematoma’ pressure gradient during craniotomy, during surgery and at the end of surgery were recorded and statistically analysed.

### Surgical methods

In the keyhole endoscopy group, the keyhole position was located according to the haematoma position, and a milling cutter formed a circular bone window of approximately 2–2.5 cm in diameter. A brain needle was used to puncture the centre of the haematoma and extract a portion of its contents to reduce ICP. A special sheath was inserted into the haematoma cavity to form a columnar working channel. Then, the endoscope was placed, suction was used to remove the haematoma, and bipolar electrocoagulation was used to stop bleeding. When eliminating a haematoma, one surgeon needed to operate with both hands: the left hand held the endoscope, and the right hand held a suction device to remove the haematoma. When bleeding was stopped by bipolar electrocoagulation, a two-person, three-hand operation mode was employed. The assistant held the endoscope, while the surgeon used the left hand to suction and used the right hand to stop the bleeding. Finally, the dura mater was sutured, and the bone flaps were attached^6^.

In the large trauma craniotomy group, a modified pterional approach was employed in the basal ganglia region, and the size of the bone window was approximately (7–8) cm × (9–12) cm. The lateral cleft was separated under a microscope to expose the cortex of the insula and form a cortical fistula. Then, in the haematoma cavity, the brain was gently manipulated with a spatula, and the haematoma was removed without touching the brain parenchyma around the lesion; bipolar electrocoagulation was then used to stop bleeding. With respect to haemorrhage on the surface of the brain lobe, such as the occipital lobe and frontal lobe, a horseshoe or coronary incision was used to perform a craniotomy according to the location of the haematoma, the haematoma was located through puncture, a cortical fistula was used to access the haematoma cavity, and the haematoma was pulled out using a brain spatula. When closing the cranium, the surgeon sutured the dura without increasing its tension and performed decompressive craniectomy based on the patient's condition^6^.

### Operation evaluation

Operation evaluation was conducted from four aspects for statistical comparative analysis, namely, operative time including total operative time, time for craniotomy and cranial closure and time for removal of haematoma, bleeding volume during the operation, the volume of blood transfusion and the haematoma clearance rate that was mainly calculated based on the 24-h postoperative CT examination (residual haematoma volume/total haematoma volume).

### Postoperative evaluation

Postoperative evaluation was reflected by four aspects: mortality rate, rehaemorrhage ratio, postoperative cerebral oedema and activities of daily living (ADL) scores. With regard to postoperative cerebral oedema, a CT scan was performed 24 h after surgery, and the largest dimension of oedema was recorded as the maximal diameter of oedema, which was denoted by D. The degree of cerebral oedema was graded as follows. Level 0 oedema: D = 0; level 1 oedema: D < 2 cm; level 2 oedema: 2 cm < D < 4 cm; level 3 oedema, D > 4 cm. In addition, level 0 oedema and level 1 oedema were regarded as mild oedema, and level 2 oedema and level 3 oedema were regarded as severe oedema. The postoperative follow-up was conducted six months after surgery, and the ADL scale was employed for evaluation of therapeutic effects. The patients with ADL grades I-III had good prognoses, and the patients with grades IV-V had poor prognoses.

### Statistical methods

The statistical package SPSS 19.0 was used for analysis. The measurement data that conformed to a normal distribution were denoted as x ± s and were compared using the *t*-test. The enumeration data were compared with the chi-square test (χ^2^). A *P* value < 0.05 was considered statistically significant.

## Results

### Changes in ICP during surgery

**Figure 1:**
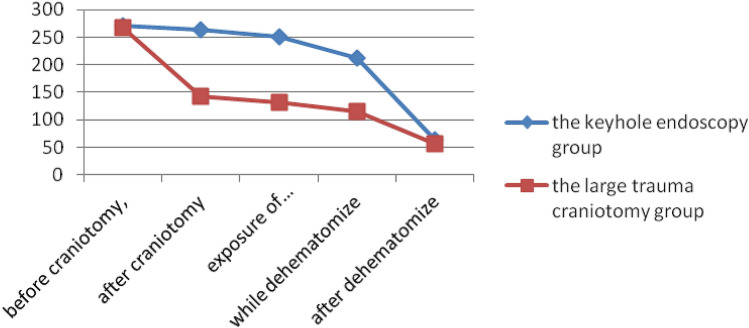
Change curves for ICP.

Before craniotomy, the mean ICP in the keyhole endoscopy and large trauma craniotomy groups was 271.3 ± 22.3 mmH_2_O and 267.4 ± 19.8 mmH_2_O, respectively, with no significant difference between the groups (*P* > 0.05). After craniotomy, ICP did not change obviously in the keyhole endoscopy group, which exhibited an average ICP of 263.8 ± 18.7 mmH_2_O, whereas in the large craniotomy group, ICP decreased significantly to an average of 142.2 ± 12.3 mmH_2_O. The difference between the two groups was statistically significant (*P* < 0.05). When the haematoma was revealed after a cortical fistula had been created, ICP gradually decreased to an average of 251.1 ± 20.6 mmH_2_O in the keyhole endoscopy group and declined to an average of 132.3 ± 10.5 mmH_2_O in the large trauma craniotomy group. This difference was statistically significant (*P* < 0.05). When the haematoma was removed, ICP gradually decreased to an average of 212.3 ± 24.3 mmH_2_O in the keyhole endoscopy group and decreased in a similarly slow manner to an average of 115.9 ± 11.7 mmH_2_O in the large trauma craniotomy group; these average values significantly differed (*P* < 0.05). After removal of the haematoma, ICP was reduced to normal in both groups, with an average ICP of 63.6 ± 9.3 mmH_2_O and 56.8 ± 8.8 mmH_2_O in the keyhole endoscopy group and the large trauma craniotomy group, respectively; there was no significant difference in ICP between the two groups (*P* > 0.05) (Fig. 1).

### Changes in the ‘brain-haematoma’ pressure gradient during surgery

The haematoma was located in the brain tissue during craniotomy. The pressures in the haematoma cavity and the brain tissue were dynamically balanced, and the pressure in the haematoma cavity was equal to the value of ICP. At this time, the ‘brain-haematoma’ pressure gradient = ICP–ICP = 0 mmH_2_O. There was no significant difference between the keyhole endoscopy group and the large trauma craniotomy group.

**Figure 2:**
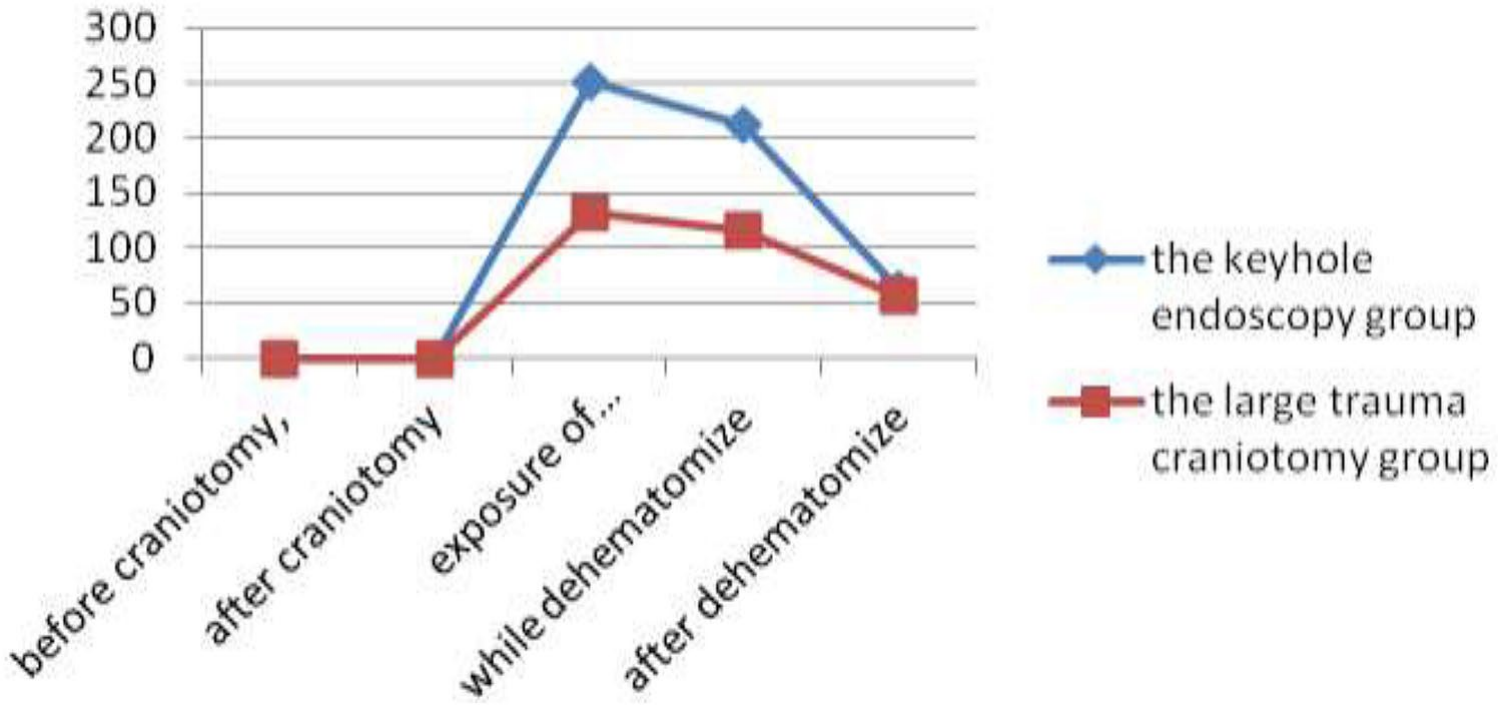
Change curves for the ‘brain-hematoma’ pressure gradient.

During the process of revealing and removing the haematoma, the ‘brain-haematoma’ pressure gradient = ICP. When the haematoma was revealed, the ‘brain-haematoma’ pressure gradient averaged 251.1 ± 20.6 mmH_2_O and 132.3 ± 10.5 mmH_2_O in the keyhole endoscopy and large trauma craniotomy groups, respectively. There was a statistically significant difference between the two groups (*P* < 0.05). When the haematoma was removed, the ‘brain-haematoma’ pressure gradient was 212.3 ± 24.3 mmH_2_O in the keyhole endoscopy group and 115.9 ± 11.7 mmH_2_O in the large trauma craniotomy group. The difference between the two groups was statistically significant (*P* < 0.05). After the haematoma was removed, the ‘brain-haematoma’ pressure gradient was 63.6 ± 9.3 mmH_2_O in the endoscopy group and 56.8 ± 8.8 mmH_2_O in the large craniotomy group, with no significant difference between the groups (*P* > 0.05) (Fig. 2).

### Surgery situation

Compared with that in the large trauma craniotomy group, the average bleeding volume was reduced in the keyhole endoscope group, as were the volume of blood transfusion, the time needed to clear the haematoma, and the mean operative time, whereas the average postoperative haematoma clearance rate was higher. (Table 1).

**Table 1:** Intraoperative comparison of the two patient groups.

Groups	mean operating time (min)	Average intraoperative blood loss (ml)	Average intraoperative blood transfusion (ml)	Average hematoma clearance rate (%)
Keyhole endoscopy group	79.5 ± 4.2	173.9 ± 12.4	0	94.8%
Craniotomy group	219.2 ± 5.8	405.6 ± 37.1	272.3 ± 14.1	83.4%
chi-square test	χ2 = 173.39 *P* < 0.01	χ2 = 305.85 *P* < 0.001	χ2 = 25.69 *P* < 0.01	χ2 = 53.97 *P* < 0.001

### Postoperative situation

Although the mortality rate and rebleeding rate were not significantly different, the average postoperative haematoma clearance rate was high, the rate of severe oedema was low, and the postoperative ADL score was better in the keyhole endoscopy group than in the large trauma craniotomy group. (Table 2).

**Table 2:** Comparison of two groups of patients in postoperative situation.

Groups	Death rate	Rebleeding rate	Severe edema rate	Rate of good prognosis in ADL
Keyhole endoscopy group	2.7%	2.7%	11.2%	86.4%
Craniotomy group	2.7%	2.7%	69.9%	38.9%
chi-square test	χ2 = 347.37 *P* > 0.05	χ2 = 347.37 *P* > 0.05	χ2 = 11.25 *P* < 0.05	χ2 = 22.37 *P* < 0.01

Common complications of intraventricular pressure monitoring include catheter dislocation, catheter blockage, intracranial haemorrhage, and intracranial infection. In the keyhole endoscopy group, 2 patients had catheter blockage, which was solved by irrigation; in the craniotomy group, 1 patient had intracranial infection, which was cured by anti-infection treatment, and 1 patient had catheter blockage, which was solved by irrigation, and there was no significant difference in the incidence of complications (*P* > 0.05, 5.6% VS 5.6%) between the two groups. Neither the death rate (*P* > 0.05, 2.8% VS 2.8%) nor rebleeding rate (*P* > 0.05, 2.8% VS 2.8%) showed any obvious changes.

## Discussion

HICH has high incidence, high mortality, and a high disability rate. Rincon et al. reported the natural mortality rate of HICH within 30 days after onset to be approximately 45%^7^. HICH can cause a series of pathophysiological changes. First, acute haemorrhage damages brain tissue, neurocytes and nerve conduction bundles, leading to brain dysfunction. Second, the increasingly large haematoma compresses surrounding brain tissue, forming pressure gradients between the haematoma and surrounding brain tissue and forcing shifts in brain tissue. Due to blocked peripheral vascular circulation and cerebral ischaemia and hypoxia of brain tissue, brain oedema occurs, causing a sharp rise in ICP and even leading to brain stem compression-induced cerebral hernia, which is life-threatening^8^. Third, the acute and chronic toxic effects produced by haematoma decomposition cause brain oedema, degeneration, and necrosis, which can further increase ICP and aggravate neurological dysfunction^9^. Therefore, in the shortest possible time, it is necessary to clear the intracerebral haematoma, relieve compression, promote the eventual return of displaced brain tissue, reduce ICP, maximize the preservation of nerve function, and create favourable conditions for the recovery of brain function^10–12^.

Currently, there are many surgical treatments for cerebral haemorrhage, most of which are empirical treatments developed within different medical centres, although no consensus has been reached to date^13^. Common surgical methods, such as trepanation and drainage, craniotomy evacuation of haematoma, and neuroendoscopic treatment for cerebral haemorrhage, have their own advantages and disadvantages and respective indications for adaptation^14,15^. Haematoma puncture and drainage are quick and simple, and they have been widely used in primary hospitals. However, this approach has disadvantages of a low haematoma clearance rate and a high rebleeding rate^16^. In addition, postoperative injection of urokinase to promote liquefaction of the haematoma requires long-term intubation and drainage, increasing the probability of intracranial infection^17^. Craniotomy for removal of haematoma involves two types of operations: large trauma craniotomy and small bone window craniotomy. The large trauma craniotomy for haematoma removal has the advantages of a high haematoma clearance rate and less chance of rebleeding, but it has more limitations, such as a long operative time, serious intraoperative brain pulling, severe secondary brain injury, heavy trauma, and more intraoperative bleeding^13^. Although small bone window craniotomy for haematoma removal relatively shortens the operative time, it can still cause serious intraoperative brain pulling and severe secondary brain injury. When ICP increases after surgery, the scope of the bone removal flap is limited, the decompression effect is not obvious, and the visual field is relatively limited; therefore, greater microscopy skill is required of the surgeon. Intracerebral haematoma removal under neuroendoscopy results in less surgical trauma and a decreased operative time. However, it is limited by neuroendoscopy. Therefore, specific surgical instruments are required to perform surgery. In general, a specific ultrasonic aspirator is used to physically break the lump and remove haematomas^14^. Due to the difficulty of mastering this technique, this procedure is difficult to perform at the majority of primary hospitals.

**Figure 3:**
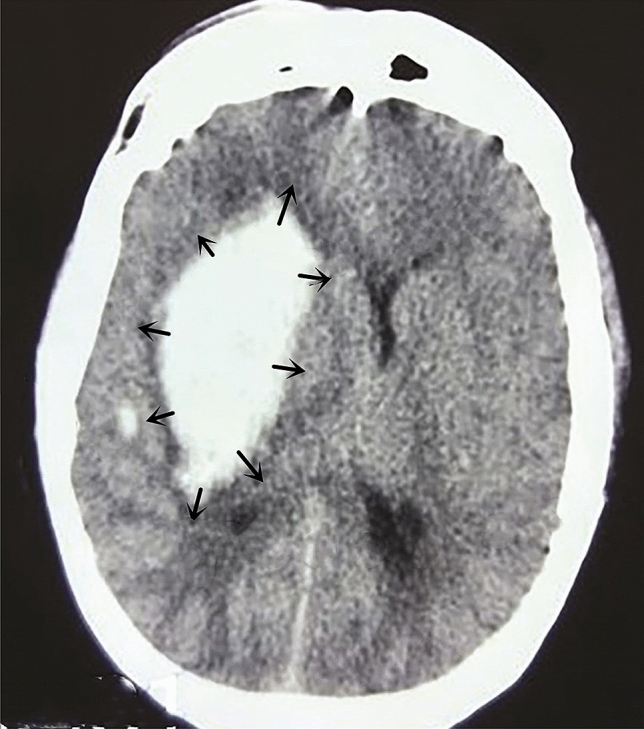
The space occupying effect of cerebral hemorrhage makes the pressure of the hematoma higher than that of the brain tissue and forces the surrounding brain tissue to shift until the pressure of the hematoma equals that of the brain tissue.

After intracerebral haemorrhage, the mass effect of haematoma caused the haematoma pressure to be higher than the brain tissue pressure, leading to displacement of the surrounding brain tissue, which eventually stopped when the haematoma pressure and the brain tissue pressure were balanced (Fig. 3). During intracerebral haematoma removal surgery, as the haematoma cavity was exposed to the environment and the haematoma was continuously being removed, the pressure in the haematoma cavity was significantly reduced, and a new reverse pressure gradient was formed between the brain tissue and the haematoma cavity. This gradient promoted the return of brain tissue to its original state and an outward shift in the haematoma that contributed to haematoma removal. However, in other surgical approaches, there are significant differences in ICP changes as well as the size and type of changes in the pressure between the haematoma cavity and brain tissue; these differences have diverse impacts on the surgical haematoma removal process and on postoperative effects.

**Figure 4:**
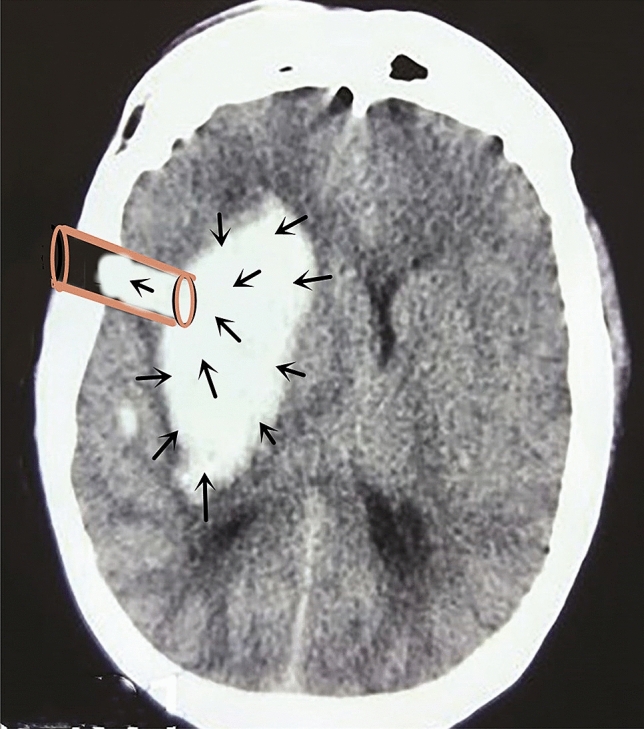
In the keyhole endoscopy group, the hematoma in the endoscopic channel has negative pressure relative to the remaining hematoma and brain tissue, thus forming a pressure gradient of “brain-hematoma-endoscopic channel”. Thus, the brain tissue further retracts, forcing the intracerebral hematoma to shift into the work channel area, and hematoma removal can be completed under synthetic action without any extraneous tissue exposure.

When an endoscopic keyhole was employed for craniotomy, ICP was not significantly reduced due to the small bone hole. However, after a cortical fistula had been created, the ‘brain-haematoma’ pressure gradient formed by surgery was large. The average ‘brain-haematoma’ pressure gradient was 251.1 ± 20.6 mmH_2_O, 212.3 ± 24.3 mmH_2_O and 63.6 ± 9.3 mmH_2_O at the start of haematoma removal, during removal and after removal, respectively, showing that the ‘brain-haematoma’ pressure difference slowly declined as the haematoma was continuously cleared and ICP slowly declined. Due to the large ‘brain-haematoma’ pressure gradient and its slow decline, haematomas in the endoscopic channel showed a sustained negative pressure state relative to other haematomas and brain tissue. Such a high sustained negative pressure contributed to the retraction of brain tissue and forced the intracerebral haematoma to move to the working channel area (Fig. 4). The haematoma was easily removed with the help of external absorption; thus, the haematoma removal rate was high, the operative time was short, intraoperative blood loss was small, and blood transfusion was not required.

Use of the keyhole technique can facilitate rapid craniotomy, remove the intracerebral haematoma with a high clearance rate in the shortest time, relieve nerve compression and minimize nerve damage. The small bone hole with a diameter of approximately 2 cm minimized the exposure of brain tissue because lack of exposure is the best way to protect brain tissue. The endoscopic channel was columnar, and the pressure was transmitted evenly around the brain. The brain tissue continued to be supported in multiple directions, and there was no obvious traction on the tissue. The damage to the brain tissue was slight; therefore, severe brain oedema did not occur after the operation. A continuously high and slowly declining ‘brain-haematoma’ pressure gradient was utilized to cause automatic flowing of the haematoma to the endoscopic channel. With the haematoma in the channel removed, the brain tissue shifted and caused the haematoma to swell further. Various endoscope angles made it easier to efficiently remove haematomas. In addition, with no need for brain pressure plates and other devices to pull the brain tissue, the keyhole technique can achieve true intraoperative microtraction with minimal damage to brain tissue, no serious brain oedema after surgery and good recovery of the postoperative patient's neurological function.

**Figure 5:**
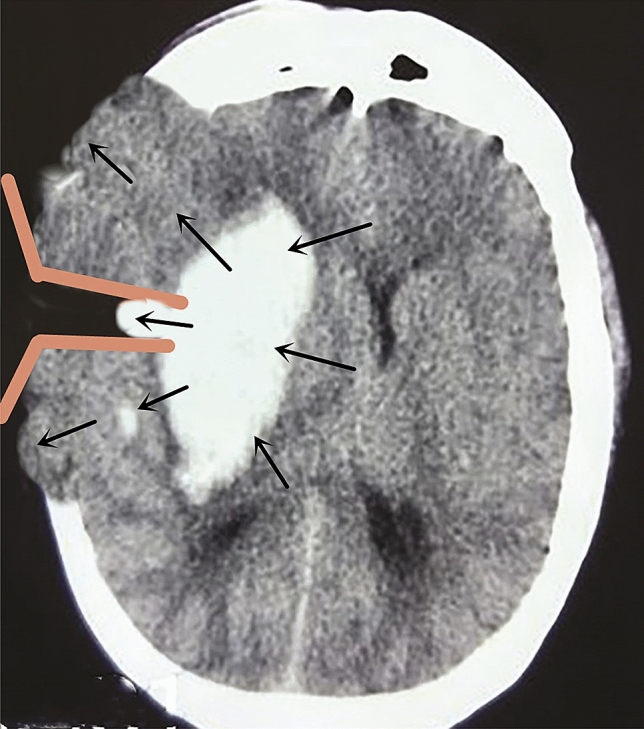
In the craniotomy group, after removing the flap and opening the dura, because of the pressure difference of “distant brain-hematoma-brain tissue around bone window”, some brain tissue bulged out from bone window under the influence of brain tissue pressure and hematoma pressure, significantly decreasing the brain tissue pressure. Thus, in the process of hematoma removal, the pressure difference of "brain-hematoma " hardly forms. The hematoma does not retract and is not easy to remove. The only way for removal is the use of a brain spatula to pull brain tissue and reveal hematoma for clearance, which is also challenging because excessive pulling can easily damage brain tissue.

After a large trauma craniotomy, ICP and brain tissue pressure significantly decreased. The brain tissue first bulged out under the influence of the ‘brain-haematoma’ pressure gradient and stopped when a balance between the brain tissue pressure and the haematoma cavity pressure was reached. Lower ICP resulted in a smaller ‘brain-haematoma’ pressure gradient after a cortical fistula had been created. The average pressure gradient was 132.3 ± 10.5 mmH_2_O at the start of haematoma removal, 115.9 ± 11.7 mmH_2_O during removal, and 56.8 ± 8.8 mmH_2_O after removal, demonstrating that ICP further decreased and the ‘brain-haematoma’ pressure gradient slowly declined as the haematoma was continuously removed. This smaller ‘brain-haematoma’ pressure gradient meant that there was little pressure on the brain tissue to shift it back to its original location and that the haematoma was not easy to retract and remove (Fig. 5). As a result, the haematoma could only be revealed and removed by pulling the brain tissue through the brain pressure plate, resulting in a long operative time, low haematoma clearance rate, massive intraoperative blood loss, and the need for blood transfusion therapy. Furthermore, excessive tension on the brain plate was likely to cause brain tissue damage, causing severe postoperative brain oedema and leading to poor postoperative neurological recovery.

Although the endoscopically assisted keyhole approach for haematoma removal has the advantages of mild brain damage and good postoperative neurological recovery, it still has certain limitations, as follows. It is necessary to accommodate the endoscope, suction apparatus and bipolar coagulation device in a relatively small space to reveal and remove the haematoma and completely stop the bleeding. Such surgery requires surgeons to have proficient micro-neurosurgery operating skills and experience with endoscopic application skills, as well as good cooperation with assistants.

In summary, the size of and variation in the ‘brain-haematoma’ pressure gradient for different surgical methods significantly influence surgical procedures and effects. ICP slowly decreased during endoscopic surgery, and the ‘brain-haematoma’ pressure gradient was large and slowly decreased. As a result, using this approach relative to large trauma craniotomy, the haematoma was easier to remove, and there was a high haematoma clearance rate, less intraoperative blood loss, a shorter operative time, and less intraoperative trauma. In addition, postoperative brain oedema was mild, and the postoperative nerve recovery rate was high. Large trauma craniotomy sharply decreased ICP, and the ‘brain-haematoma’ pressure gradient was small. Brain tissue retraction was poor in this case; therefore, the haematoma was difficult to clear, and it was necessary to use a brain spatula to pull the haematoma to remove it. In addition, the surgery situation and postoperative situation showed a relatively low haematoma clearance rate, massive intraoperative bleeding volume, long operative time, large intraoperative trauma, severe postoperative brain oedema, and a low postoperative nerve recovery rate. However, the number of patients in this group was still relatively small, and a large sample will be required for further comparative studies.

## Abbreviations

HICH: Hypertensive cerebral hemorrhage
ADL: Activity of daily living
